# The datafication of reproduction: time‐lapse embryo imaging and the commercialisation of IVF

**DOI:** 10.1111/1467-9566.12881

**Published:** 2019-10-10

**Authors:** Lucy van de Wiel

**Affiliations:** ^1^ Department of Sociology University of Cambridge Cambridge UK

**Keywords:** reproductive health, reproductive rights, reproductive technology, IVF, time‐lapse embryo imaging, datafication

## Abstract

The 21st century has witnessed the emergence of *in silico* reproduction alongside the familiar *in vitro* reproduction (e.g. IVF), as increasingly large and automatically‐generated data sets have come to play an instrumental role in assisted reproduction. The article addresses this datafication of reproduction by analysing time‐lapse embryo imaging, a key data‐driven technology for embryo selection in IVF cycles. It discusses the new forms of knowledge and value creation enabled by data‐driven embryo selection and positions this technology as a harbinger of a wider datafication of (reproductive) health. By analysing the new ways of seeing embryos with ‘*in silico* vision,’ the ‘data generativity’ of developing embryos and the patenting of embryo selection algorithms, I argue that this datafied method of embryo selection may not just result in more or less ‘IVF success,’ but also affects the conceptualisation and commercialisation of the assisted reproductive process. In doing so, I highlight how the datafication of reproduction both reflects and reinforces a consolidating trend in the fertility sector—characterised by mergers resulting in larger fertility chains, online platforms organising fertility care and expanded portfolios of companies aiming to cover each step of the IVF cycle.

Recent years have seen an intensification of the datafication of reproduction, as increasingly large and automatically generated data sets have come to play an instrumental role in the technological reproduction of human life. This development is evident at all stages of the reproductive process, whether in fertility apps for timing conception, genomic fertility testing or the use of quantified visual data for embryo selection in IVF (*in vitro* fertilisation). The emergence of *in silico* reproduction alongside the familiar *in vitro* reproduction (e.g. IVF) follows from an understanding that an increasing number of aspects of reproduction can be ‘measured and monitored and treated as technical problems with technical solutions’ (Kitchin 2014, p. 181). It concerns not simply the use of data, which has long been part of technologised reproduction, but the attempted optimisation, automation and standardisation of assisted reproductive processes in ways that rely on, generate and analyse large data sets with novel computational technologies, including predictive analytics and machine learning.

One of the major changes in assisted reproduction today is the introduction of data technologies in the fertility lab with the aim of improving, automating and standardising previously manual processes. Yet in spite of the growing scholarship on digital health, the datafication of assisted reproduction remains surprisingly understudied. As the so‐called ‘data revolution’ is transforming health care at large (Kitchin 2014), it is pertinent to focus on assisted reproduction in particular because it is a relatively unregulated sector in which bioinnovations – including data‐centric ones – are introduced rapidly. Such innovations are relevant beyond their clinical (in)efficacy given the culturally specific and politically charged nature of reproductive processes and their reconceptualisation and reconfiguration in the face of new reproductive technologies (Franklin 2013).

One key influential reproductive data technology is time‐lapse embryo imaging, a new data‐intensive method of embryo selection used for deciding which embryos will be implanted in the womb in IVF. This embryo selection method displaces the embryologist's manual appraisal of embryos under the microscope by continuously filming them in the incubator, quantifying the visual information and predicting their viability through algorithmic analyses. Hailed as the ‘greatest breakthrough in IVF in 25 years,’ and criticised for its rapid introduction in the absence of ‘high level evidence of improved live birth rates, safety and cost effectiveness’ (Armstrong *et al*. 2015a,b, Harper *et al*. 2017, Walsh 2013a), time‐lapse embryo imaging is now widely available in fertility clinics worldwide and is changing the face of IVF.

Major players in the fertility industry – including pharmaceutical giant Merck and biotechnology company Vitrolife – have heavily invested in time‐lapse imaging and distribute systems to clinics across the globe. Although the HFEA, the UK's fertility regulator, advises that there is ‘certainly not enough evidence to show that time‐lapse imaging improves birth rates,’ demand for this technology is growing (HFEA 2018). For example, Vitrolife reported almost $100 million net global sales of time‐lapse technologies in 2017, while the first quarter of 2018 has already secured $30 million net sales after the company received regulatory approval for its *Embryoscope* system in China, one of the world's largest fertility markets (BusinessWire 2011; Vitrolife 2018a). As fertility clinics invest in time‐lapse imaging, more intended parents will be confronted with the question of whether to include this add‐on in their IVF cycles – often at an increased cost.

This article focuses on the new forms of knowledge and value production emerging with this data‐driven time‐lapse method of embryo selection and situates them in the techno‐economic dynamics of an emerging global reproductive data infrastructure. It is written from the conviction that, in order to understand contemporary socio‐technological changes in human reproduction, we have to take into account the rationalities, power relations and global institutional configurations in the ‘reproductive‐industrial complex’ from which these bioinnovations emerge (Vertommen 2017). In doing so, this project seeks to not simply present a case study of one particular reproductive technology, but approaches the rise of time‐lapse embryo imaging as a lens onto the dynamics underlying the introduction of data‐driven bioinnovations in (reproductive) health care more broadly.

Adopting Vertommen's genealogical method for the analysis of emerging bioeconomies, this article characterises the datafication of reproduction by analysing the genealogy of data‐driven embryo selection in the contemporary global fertility sector (2017, pp. 286–7). Through a case study approach, which Feagin *et al*. (1991, p. 1) define as an ‘in‐depth, multifaceted investigation [that is] conducted in great detail and often relies on the use of several data sources,’ I approach time‐lapse selection as ‘an instance of [the] broader phenomenon’ of datafied reproduction. Transposing Bal's (2002) method of cultural analysis to this critical case study, I analyse time‐lapse embryo imaging by close reading key discursive objects that organise the marketisation of this technology – including patents, financial reports, direct‐to‐consumer advertisements and online platforms – in dialogue with influential conceptual frameworks in reproductive sociology and critical data studies.

This study builds on the work on the commercialisation and conceptualisation of prenatal life and fertility technologies in the field of reproductive sociology, notably by scholars such as Sarah Franklin (2013), Charis Thompson (2013) and Marcia Inhorn (2015). It provides an update on Deborah Spar's (2006) analysis of the fertility market in *The Baby Business* by focusing on a newer, data‐driven technology that allows for a characterisation of more recent changes in the IVF sector, including its ongoing expansion and consolidation, the rise of private equity investments, the significance of patenting and the commercialisation of reproductive data. These focus points align with a surge of scholarship in science and technology studies concerned with the relation between reproduction, cellular life and capital. Notably, the work on ‘biovalue’ and ‘biocapital’ by Sarah Franklin (2013), Catherine Waldby and Robert Mitchell (2006), Cori Hayden (2003) and Sunder Rajan (2006) analyses the transformation of biological substance into ‘generative forms of capital through which further commodities and value are created’ (Murphy 2017, p. 13). Drawing on Melinda Cooper's characterisation of the self‐generative capacities of stem cells and finance, it analyses the generative relations between embryogenesis, capital and biodata emerging with data‐driven IVF (2008). It brings this work into dialogue with data studies by drawing on Van Dijck and colleagues' (2016), Hogle's (2016) and Kitchin's (2014) critical readings of the governance implications of ‘datafication.’ In doing so, it introduces in the field of reproductive sociology a focus on the ‘data revolution’ in global IVF, which is fundamentally changing the mechanisms through which we can conceive, control and commercialise reproduction at large.

In this way, I examine how the development and distribution of server‐connected embryo selection apparatuses is remaking assisted reproduction by introducing ‘emergent reproductive data infrastructures,’ ‘new reproductive bioeconomies of data sharing’ and an ‘expansion of the scope of reproductive risk through predictive analytics’ by focusing on three aspects. Firstly, I propose reproductive data technologies such as time‐lapse embryo imaging introduce an ‘*in silico* vision,’ an algorithmic way of seeing through historical data sets that makes previous populations visible as the basis for future predictions. Secondly, I examine several controversies surrounding the patents and proprietary algorithms of embryo development and discuss how they reveal an ongoing renegotiation of the locus and ownership of expert knowledge and medical authority. Thirdly, by situating this technology in the institutional context of consolidating fertility, biotech and pharmaceutical companies, I address how time‐lapse embryo imaging brings together self‐ and automated tracking, data infrastructures and social media in contemporary practices of technologically assisted reproduction. In doing so, I argue that this datafied method of embryo selection may not just result in more or less ‘IVF success,’ but also affects the conceptualisation and commercialisation of the assisted reproductive process and impacts the very coming into being of prenatal life.

## *In silico* vision

Since its 2013 introduction in the UK, time‐lapse embryo imaging has been promoted by major fertility clinics as an alternative, and superior, form of embryo selection. Whereas conventional selection relies on once‐daily assessment of *in vitro* embryos under the microscope, time‐lapse embryo imaging enables the embryos to remain undisturbed in the incubator and be photographed every 5–20 minutes. The visual data derived from these images are matched against predictive parameters to assess embryo quality. Emerging in the wake of an increasingly public visual interface with prenatal life, e.g. through imagery of IVF and fetal ultrasound, both of which have had a profound impact on public and private imagination of the reproductive process, the resulting time‐lapse embryo videos add yet another visual dimension to the encounter with early human life on screen (Duden 1993, Franklin 2013). For example, a downloadable embryo video is part of the IVF package at CARE, the UK's largest fertility group, and Genea clinics live‐stream images of developing embryos from the incubator to intended parents’ iPads. Yet beyond another mode of medical imaging that brings the embryo into view, these embryo videos introduce an ‘*in silico* vision,’ an algorithmically assisted way of seeing that makes the embryo legible, and its viability calculable, in new ways.

The advent of routine time‐lapse imaging in the fertility clinic reconfigures the visual recognition of embryonic developmental stages. Rather than requiring manual observation, automated tracking algorithms record a number of visual aspects of development (e.g. cell division or cell quantity). In this process, a multiplicity of parameters recording specific visual aspects of the cells may be combined to ascribe a unique quantified value to each embryo (Merck 2015). In turn, the time‐lapse system matches the resulting visual data against historical data about previous cohorts of embryos to give a prediction of embryo viability. The viability outcomes are automatically layered onto the embryo videos with numerical scores, colours or superimposed words (‘high’ or ‘low’). Data analysis thus plays a key role in watching embryos in time‐lapse embryo imaging as a means of not only making them visible, but rendering their viability legible, calculable and manageable.

The move from daily examinations under the microscope to time‐lapse embryo imaging then introduces a data‐assisted way of seeing, or ‘*in silico* vision’ in the embryological work flow. Now touching the screen instead of the petri dish, time‐lapse embryo imaging allows embryologists to observe, record and compare cellular behaviours that occurred in the petri dish in their absence – both in the incubated embryo cohort and those historical populations that preceded them. Tracking algorithms can record key developmental markers and provide suggestions for manual annotation (Vitrolife 2015a). They likewise detect activities ‘beyond what the human eye is capable of measuring’ by recording, for example, changes in the embryo's textural granularity (Merck 2015). In each of these ways, the introduction of *in silico* vision into the IVF lab presents an alternative mode of ‘authorised seeing’ alongside the embryologist's medical gaze through the microscope (Foucault 1973, Jasanoff 2017).^1^

With *in silico* vision, ‘assisted seeing’ through data correlations integrates calculation and observation with the aim of detecting regularity in temporally and spatially disperse embryo cohorts. This approach entails an epistemological shift in knowledge production about embryos, which is becoming a new standard as the popularity of these time‐lapse systems grows.^2^ The marketing of time‐lapse imaging positions *in silico* vision as a superior method of non‐invasive embryo observation that allows for ‘a more objective analysis’ (Vitrolife 2018b). Although the epistemic fallacies underlying such claims to observational objectivity have been demonstrated,^3^ I here follow Sheila Jasanoff's approach of not attempting an ‘inquiry into the validity of particular data claims,’ but exploring ‘how power works in re‐representing things that happen in the world in the form of data points’ (2017, p. 2). The claim to a ‘more objective analysis’ is in line with what Jasanoff calls a ‘view from nowhere’ – in contrast with views from everywhere and somewhere – as the ideal‐type of a ‘disinterested purity of science.’ This regime of seeing aligns with the problematic notion that ‘data’ can ‘sanitize the world of observation,’ erasing from view the observational standpoints and associated political choices underlying the compilation of authoritative information (Jasanoff 2017, p. 12).

However, as manual observation and assessment is supplemented by automated and datafied methods, there is not an erasure, but in fact a multiplication of observational standpoints as the embryologist ceases to be the sole observer and decision‐maker in embryo selection. *In silico* vision denotes a more networked model of knowledge production that creates new forms of dependency in attaining ‘more objective’ visions of prenatal life that are enmeshed with attendant data‐centric forms of commodification. Essential components of *in silico* vision – the system itself, its interfaces, the data sets it generates and the algorithms with which they are analysed – may now be owned by corporate actors, thereby positioning embryo selection as a significant nexus of power relations and capital flows in contemporary IVF.

This paper examines the power relations at work in the establishment of *in silico* vision and the widespread introduction of data‐driven embryo selection, as a particular organisation between those that produce, analyse and claim ownership of embryo data is built into the very means of seeing prenatal life. These concerns are vividly articulated through the controversies surrounding the patenting of data‐driven embryo selection systems.

## Patenting data‐driven embryo selection

The patenting of time‐lapse imaging systems brought embryo development into the realm of private property and thereby provoked controversy in the scientific and bioethics community. Because these patents include temporal parameters of embryo development, they provoked discussions about the patentability of natural phenomena. This section addresses the institutional and regulatory contexts from which the patenting of embryonic development emerges and their significance in rendering embryo development generative and valuable in new ways. It thereby highlights how the datafication transition in IVF raises fundamental questions about the conceptualisation and commercialisation of prenatal life as data‐driven embryo selection becomes a means for creating new forms of capital accumulation.

Between 2011 and 2013, both US and European patent offices issued patents covering the timing of cellular development as ‘predictive parameters’ in embryo selection to Stanford University, with exclusive licensing to Auxogyn (now Progyny), the company that produced the *Eeva* test. The patents describe the association of ‘good developmental competence’ with cellular temporal markers, such as a ‘duration of first cytokinesis […] between 0 and 30 minutes’ and a ‘time interval […] between the resolution of cytokinesis 1 and the onset of cytokinesis 2 [of] 8–15 hours’ (Baer *et al*. 2011, Wong *et al*. 2012, 2013). Rather than only describing the technicalities of the embryo selection method, the patents thus also include the temporal specifics of embryo development as part of the patented intellectual property.

Consequently, the question arose to what extent the timing of embryo development is a natural phenomenon, and therefore unpatentable, or an essential part of a new, patentable invention. Jacques Cohen, chief Editor of *Reproductive BioMedicine Online*, led a scholarly response to the Auxogyn patents and wrote a plea against ‘patenting time and other natural phenomena’ in this journal:

Claiming cell cycle timing or duration as an invention that merits a patent would strike most students of developmental biology as an unlikely proposition but researchers at Stanford University have successfully done exactly that! The first three cell cycles in the human embryo developing *in vitro* are now owned by a corporation. (Cohen 2013a, p. 109)

He argues that ‘the length of the cell cycle is not an invention and its key role in development is not a new observation; it is an indisputable and well‐known fact of nature’ that has been described since the late 19th century. A precedent of patenting temporal phenomena, he claims, will have long‐term problematic effects (2013a, p. 109). In response, Stanford professor and inventor of the patent Renee Reijo Pera claimed that the patents cover the ‘assays intended to distinguish optimal [and suboptimal] embryos for transfer in IVF’ and therefore entail a method rather than a natural phenomenon (2013).

In the ensuing riposte between Reijo Pera and Cohen, the former argues that the ‘diagnosis of embryo viability’ does not address a ‘naturally occurring phenomenon’ because there is ‘no need to distinguish quality amongst as many as 5–10 embryos (or even more) in natural conception; and in nature women simply do not conceive outside of the body’ (2013, p. 113). Cohen responds that no studies have supported the premise that *in vivo* and *in vitro* cell cycles are fundamentally different processes; in fact it is their close resemblance that has resulted in the birth of over 5 million children from IVF. ‘Arguing that those processes were somehow not natural (and therefore patentable),’ he suggests, ‘may instigate an entirely different discussion, not unlike those that engaged the opponents of IVF in its early days’ (Cohen 2013b, p. 115).

The uncertainty surrounding the patentability of embryo development in the context of time‐lapse embryo imaging is another iteration of an ongoing renegotiation of legal ownership of human biological substances, and the data derived from them. In *Bioinformation* (2017), Bronwyn Parry and Beth Greenhough describe the difficulties of differentiating between ‘discoveries’ from ‘inventions’ in the context of biotechnologically reworked material, such as isolated DNA or immortalised cell lines. A series of legal rulings have determined that intellectual property rights to human biologicals can be claimed even if they are derived from human bodies, ‘because bringing them into the world was deemed an act of manufacture or invention, not just discovery’ and the patents would recompense corporations for their expended labour (Parry and Greenhough 2017, p. 74).

Yet it is important to recognise that in the case of time‐lapse embryo imaging, the legal establishment of embryo development as patentable property occurs in response to purportedly ‘non‐invasive’ data‐driven technologies. Time‐lapse embryo imaging does not isolate or reconfigure the embryo in any way that is directly instrumentalised – as is the case for previously patented synthesised DNA or immortalised stem cell lines. Rather, it introduces new ways of extracting and analysing data from embryos and thereby repositions certain temporal parameters of embryo development as instruments for selection. It is, then, its manifestation in time as potential data points that can be instrumentalised in algorithm development and algorithmic analysis that de‐naturalises and re‐technologises the embryo and its divisions. We may consider how the broader trend of datafication in health care, and the attendant patenting of data‐driven health technologies, has re‐ontologising effects, as it brings more previously natural bodily phenomena in the realm of patentable inventions by virtue of their changing relationships to the expanding data sets and algorithmic instruments that record and analyse them.

Returning to Pera‐Reijo and Cohen's discussion, the latter's response does not address another, possibly more interesting aspect of Pera‐Reijo's justification of patenting the embryonic cell cycle, namely the political bioeconomies of embryo research and its clinical translations. Pera‐Reijo explains the patent application followed the Republican 1996 Dickey–Wicker Amendment, which ceased US federal funding for human embryo research that resulted in the destruction of embryos, including those donated by IVF patients (2013).^4^ This restriction has resulted in scientists such as Pera‐Reijo searching non‐federal funding from for‐profit partners, who would invest private capital in embryo research that could be translated into clinical benefits. She states that ‘without patents to protect the inventions made in this process, it would be nearly impossible to attract the investment finance needed to move a technology from the research and development phase, through clinical trials, through the regulatory process and ultimately to commercialization’ (Reijo Pera 2013, pp. 113–4). Conservative politics, informed by a widespread anti‐abortion sentiment in the United States, thus played a key role in enlisting embryo research within a capitalist logic that requires a redefinition of embryo development as invention.

Beyond the question of whether the patent is legitimate, what is at stake in these developments is the marketisation of evolving data and algorithms used for data‐driven embryo selection. While Cohen's critique focused primarily on fixed temporal specificities of prenatal development, these and later time‐lapse imaging patents describe temporal markers as more dynamic variables. For example, the same patent quoted above identifies ‘first cytokinesis, the second cleavage division and synchronicity of the second and third cleavage divisions’ as parameters that

can be measured automatically using the cell tracking algorithms and software previously described. The systems and methods described can be used to diagnose embryo outcome with key imaging predictors and can allow for the transfer of fewer embryos earlier in development. (Wong *et al*. 2013)

Rather than fixing specific temporal values of these parameters, it is precisely a more dynamic model which combines ongoing automated tracking, data generation and algorithmic analysis that underlies a data‐driven approach to ‘diagnos[ing] embryo outcome with key imaging predictors’ (Wong *et al*. 2013). Reframed as diagnosis, this claim to predicting embryonic developmental potential is highly marketable in an IVF process that is characterised by uncertainty at each step of the way.

Yet the key transformative aspect of this dynamic, data‐driven embryo selection remains underdiscussed in the patenting debate; it follows less from questions on the nature of the embryo and more from time‐lapse systems’ introduction of the *data‐generativity* of embryos. Cellular generativity was at the heart of the patenting of stem cells, through which, Cooper argues, ‘the self‐regeneration of life will coincide with the self‐valorisation of value’ (2008, p. 147). The patenting of embryo development similarly points to a mode of cellular generativity that coincides with value production. The incubated embryo's data‐generativity propels not only clinical and scientific knowledge production, but also the emergence of future life, as the data flows drawn from time‐lapse embryos can be repurposed as tools for future selections. Once datafied as both tool and object of selection, the incubated developing embryo enters the realm of economic valuation neither, in the first instance, as an exchangeable commodity, nor as the materially and commercially self‐accumulative stem cell line (Cooper 2008, p. 148), but rather as a generative node in an ongoing automated process of data and algorithm production that anticipates and, potentially, enables future reproduction.^5^ In other words, what is at stake in the datafication of embryo development is the transmutation of enmeshed knowledge, reproductive and capital value emerging from the data‐generativity of prenatal life.

## The algorithmisation of embryo development

At the heart of the valuation of data‐driven embryo selection is the emergence of new software and algorithmic products, which produce new forms of datafied biocapital. As Pottage remarks, it has not only been patenting but ‘the adroit exploitation of trademarks and branding strategies,’ that has spurred the growing popularity of particular time‐lapse embryo imaging systems. Of all the embryology technologies used in fertility labs, time‐lapse embryo imaging is one of the few that is branded and directly marketed to patients (Pottage 2018). The specific software and algorithms developed on the basis of the data‐generativity of previous embryo populations have moreover become products in their own right that are integral to the knowledge production and commercialisation strategies in data‐driven embryo selection.

The datafication of embryo selection converts numerous variables of embryo development into quantified data and this phenomenon enables the emergence of new reproductive bioeconomies of data sharing between different actors and institutions in the fertility sector. In the field of critical data studies, concerns have been raised about the ‘big data divide,’ or the ‘exacerbation of power imbalances in the digital era resulting from the differential access to data.’ Critical reflection on datafication, it is suggested, requires considering ‘the asymmetric relationship between those who collect, store and mine large quantities of data, and those whom data collection targets’ (Andrejevic 2014, p. 1673).

When IVF cycles become data‐generating, different organisations in the fertility sector become data‐rich in new ways. When using these technologies, the fertility clinic takes up a new role of gathering sizeable data sets on developing embryos in routine clinical practice and, in some cases, using these to develop in‐house algorithms. While many clinics have R&D activities, the ongoing data collection at the scale that time‐lapse systems introduce is unprecedented, as is the introduction of algorithmic products to render this data legible. Time‐lapse system producers, likewise, are gaining access to uniquely large, privately held data sets about embryo development. For example, Vitrolife, producer of the popular *EmbryoScope* system, has access to embryo development profiles and implantation outcomes from over 30,000 embryos. Embryologists and IVF clinics worldwide have contributed to this data set since 2009, thus reportedly creating ‘the world's largest database of embryo development with known clinical outcome’ (Montag 2015, Vitrolife 2015a). Emerging from a market‐driven context, time‐lapse embryo imaging systems are thus instrumental in the creation of asymmetric relations of private reproductive data ownership, diverging significantly from a public health‐approach to open data sharing that characterised the early history of IVF in the 1970s and 1980s.

Rather than inherently valuable, these large‐scale data sets extracted from developing embryos only acquire value when ‘data are collated, curated, interpreted and otherwise acted upon’ (Lezaun 2013, p. 481). This work of rendering embryo data valuable in both reproductive and monetary terms is one of the new forms of labour emerging with datafication that becomes visible through the marketisation of algorithmic software for embryo selection. CAREfertility, the UK's largest fertility group, for example, has developed its own proprietary algorithms for embryo selection using the *Embryoscope*. Beyond the potential for improving reproductive outcomes in their reproductive cycles, this process itself has become part of its communication to patients:

Our scientists are world‐leaders in time‐lapse technology, and our CAREmaps technique is really highly developed; we've innovated models that can help us choose the best embryo more reliably, allowing us to see whether each has a low, medium or high chance of success. (CAREfertility 2018)

Rather than only promoting time‐lapse embryo imaging itself, the CARE website specifically markets its proprietary CAREmaps (morphokinetic algorithms to predict success) as the key to IVF success. The datafication of embryo selection thus creates novel algorithmic products, which reflect both the new forms of bioinformatical labour in the fertility clinic and new forms of value production both through the branding of technological innovation and a promise of increased IVF success that comes with an additional price tag.

At the level of the producer, the labour of collecting and instrumentalising embryo data likewise yields software products. Vitrolife's ‘largest database’ of known implantation data (KID) is translated into a valuable asset through its *KIDScore* tool. Along with Vitrolife's *Embryo‐Scope*, clinics can purchase this software package, which consists of algorithms that measure the ‘implantation potential’ of the embryos in the incubator and provides a ‘morphokinetic score’ between 1 and 5 to embryologists, who can then ‘select the embryos ranked high with better chances of implanting and becoming a child’ (Vitrolife 2015b). The rival system *Eeva* similarly is coupled with the *Xtend Algorithm* software package, which was developed on the basis of multi‐centre reference data on file at producer Progyny (Merck 2015). *KIDScore* and *Xtend Algorithm* assign scores to the embryos to indicate which is more likely to survive. Given that these algorithmic tools rely on large sets of known implantation data, this practice newly aligns the generation of biodata with the generation of biocapital. Social scientist Linda Hogle argues that a ‘tidal wave of efforts to extract value from health data has accompanied the big data phenomena, leading to considerable investments by pharmaceutical, medical device and health risk management companies’ – and, in this case, leading to algorithmic products in their own right (2016, p. 386).

The development of Vitrolife's and Merck's algorithmic products relies on the presence of existing networks of data connectivities between pharmaceutical, biotechnological and fertility companies, given that *KIDScore* and *Xtend* were developed on the basis of data sets sourced from IVF clinics across the world. The (contested) claim that such networked embryo data collection is feasible with these time‐lapse systems is itself a key element in the marketisation of these algorithmic products. After all, their selling point is not simply the promise of improved pregnancy rates, but an improved workflow in the lab. Vitrolife emphasises that *KIDScore* is easy to use and requires annotation of only a limited number of variables, which its predictive analytics method anticipates. It thereby enables a ‘high level of consistency in embryo scoring in your clinic’ (Vitrolife 2015b). Echoing Jasanoff's (2017) ‘view from nowhere’ regime of sight, this discursive framing of the software points to ‘an overarching principle of interchangeability’ underlying the promise of datafication in IVF, which applies not only to intra‐clinic, but also inter‐clinic variability (Lezaun 2013, p. 481). It is this principle that motivates the claim that ‘KIDScore is universal to all clinics and can be used immediately without acquiring your own data first’ (Vitrolife 2015b). The upholding of a model of universality and interchangeable standardisation is both a key driver and an effect of the datafication of embryo selection. Similarly, it is part of a marketing strategy to extend automated embryo selection to more clinics, while being in and of itself a condition of emergence for the networked reproductive bioeconomies of data sharing and data ownership emerging with data‐driven IVF.

As a result, the datafication of embryo selection entails at once the clinical introduction of integrated apparatuses for reproductive data generation, the creation of connected networks of data sharing, and the production of biocapital out of biodata by means of algorithmisation – all of which combine in a system that is marketed directly to patients and fertility clinics. The large‐scale redistributions of embryo data between fertility companies that produce and use time‐lapse embryo imaging not only create new forms of value, but also reorder institutional relationships as lines between research and clinical practices are blurred. What is at stake in these developments is that data asymmetries between clinical, pharmaceutical and biotechnological organisations reflect and reconfigure the power dynamics in the fertility sector, which I will discuss in the next section.

## Consolidation and reproductive data infrastructures

The datafication of reproduction is not only transformative in its own right, it is but also indicative of how the broader IVF market is being reshaped. The growing popularity of time‐lapse embryo imaging is situated within an expanding, and increasingly consolidating, fertility sector. The move towards consolidation is manifest in the merging of fertility clinics into larger chains, the growing influence of online platforms in organising fertility care and the portfolio expansion of pharmaceutical and biotechnological companies to include a wider range of fertility products and incorporate each step of the fertility journey. The institutional genealogy of time‐lapse embryo imaging gives insight into, and emerges from, these consolidating developments in the global fertility industry.

### Consolidating fertility groups: mergers and acquisitions

As the global fertility market is growing steadily and is estimated to exceed $21 billion by 2020, fertility clinics are increasingly merging into larger chains (Maida 2016). Trends of reproduction later in life, greater awareness and acceptance of fertility treatments, increasing privatisation in the UK and increasing insurance coverage in the US have been suggested as drivers in this expansion (De Martino and Shapiro 2017, Williams *et al*. 2017). Whereas new freezing technologies (e.g. egg freezing) preserve reproductive potential and expand IVF's target group with fertile women, new data technologies predict reproductive potential and expand the IVF cycle with additional treatments (add‐ons such as time‐lapse embryo imaging).

The growth in the sector has been characterised by an ongoing ‘merger and acquisition cycle’ as large fertility groups expand their international reach. For example, Australian market leader Virtus Health, the world's first publically listed fertility business, operates 46 IVF clinics, after having completed four acquisitions in 2016–2017 and expanding to Ireland, Denmark and Singapore. Likewise, Abu Dhabi‐based NMC Health acquired both EUVITRO in Spain in 2015 ($162 million) and Fakih IVF Group in the United Arab Emirates ($207 million) (Williams *et al*. 2017). In the UK, CARE Fertility is the largest provider of IVF and has a steadily expanding chain of fertility clinics across the country and in Ireland (CHR 2015). The 2017 merger of Spanish IVI and US RMNAJ has created the world's largest fertility group, reaching around €300 m revenue (Pedrós and González 2017).

The datafication of reproduction is situated within these consolidating developments in the fertility sector. Given the significant price tag of time‐lapse embryo imaging systems, transitioning to this data‐driven method of embryo selection is more feasible for larger clinics. Aided by economies of scale, larger, consolidated clinics are typically early adopters of these high‐investment systems. For example, abovementioned Fakih IVF announces on its homepage that it was the first to introduce the *EmbryoScope* in the AUE. As Madeira and Carbone (2016, p. 112) report, the high cost of lab equipment is frequently mitigated through group discounts if they are purchased by a larger fertility organisation. Likewise, CAREfertility (2018), the largest UK fertility group, writes in a large header on its website that they ‘were the first UK clinic to introduce time‐lapse embryo imaging.’ Promoting these technologies to (potential) patients and the wider public, CAREfertility was at the centre of high‐profile media exposure of time‐lapse embryo imaging, which included televised BBC news reports on purportedly the ‘biggest breakthrough in IVF in 25 years’ (Walsh 2013b).

This association between consolidation and high‐investment innovations gains another dimension in the context of datafication. Because time‐lapse imaging generates data streams with each IVF cycle, larger centres with more annual cycles have the benefit of gaining larger data sets on embryo development. Depending on whether this data is thought to be clinic‐specific or sufficiently standardised to be comparable amongst different branches, these data sets can attain biovalue as a means to do research and develop algorithms for embryo selection derived from in‐house IVF cycles.

By rendering the IVF cycle both data‐driven and data‐generative, time‐lapse embryo imaging introduces an infrastructural change in the organisation of assisted reproduction. Beyond adding another ‘add‐on’ to an increasingly wide array of treatment options for IVF patients, the server‐connected time‐lapse imaging systems establish a wider reproductive data infrastructure to facilitate embryo selection in which the machines function as generative nodes. No longer confined within the walls of the IVF lab, embryo selection becomes a process that is differently dispersed across time and space as collected embryo data may be shared with patients, embryologists or manufacturers. The practices of data sharing across these new infrastructures differ as some clinics only use a local area network and keep their data in‐house, while others share and receive embryo data with other organisations. The new pathways for (automated) embryo data sharing that emerge with the introduction of time‐lapse embryo imaging enable new forms of connectivity between actors in the fertility industry – whether through the live‐streaming of embryo videos from the incubator to the intended parents’ iPad or through downloads of updated parameters for embryo selection from the manufacturer into the local time‐lapse system. Even if not all pathways for data sharing built‐in to the system are necessarily in use, the introduction of time‐lapse systems facilitates automated embryo data exchanges between the manufacturer, the patient and the incubator in the IVF lab. The resultant key shift is that the direction and scope of embryo data flows are constrained by the clinic's decision‐making rather than primarily logistic in nature.

The spatial dispersal of the embryo selection process enabled by time‐lapse systems particularly suits the spatial dispersal of consolidated fertility companies that expand their geographical reach through mergers and acquisitions. The connectivity built‐in to the time‐lapse systems offers a means of bridging the distances between clinics within a single group by streamlining embryo selection protocols and practices and by sharing embryo data to build proprietary data sets. The alignment of in‐house reproductive data infrastructures and the organisational model of consolidated fertility companies may thus widen the gap associated with data and financial asymmetries between smaller and larger organisations. In the case of time‐lapse embryo imaging, consolidation and datafication thus appear to function as mutually reinforcing conditions of co‐emergence. Larger clinics facilitate the introduction of the apparatuses while the connected and automated method materialised in the machine facilitates coordinating clinical processes across different labs and clinics.

### Platformising fertility: consolidating across technological domains

The institutional genealogy of time‐lapse embryo imaging highlights a wider trend of ‘platformisation’ (Van Dijck *et al*. 2016). Here the online fertility platform, rather than the fertility clinic, comes to function as a key organising principle of fertility care. A case in point is the *Eeva* test, which was first produced by Auxogyn, a biotechnological company which attained exclusive licensing for the technology through the abovementioned Stanford patent. In 2014, Auxogyn merged with *FertilityAuthority*, a ‘patient‐matching technology platform’ and self‐reportedly the world's largest fertility web portal with 1 million monthly visitors, which had itself acquired the leading global FertileThoughts.com social network in 2010 (Fertility Authority 2015). The resulting Progyny, *Eeva's* producer and self‐described ‘digital health company,’ is organised around the online platform as the point of access to a network of clinics and a variety of in‐house services, including IVF Advantage (fertility loans), Eggbanxx (egg freezing) and Progyny corporate fertility insurance.^6^ In this platformised approach to fertility care, Progyny's mission is to be ‘the go‐to source for all fertility solutions’ (Mack 2016). It thus brings digital health to assisted reproduction by combining investments in a data‐driven embryo selection technology with a digital reproductive health platform that integrates branded biotechnological, clinical, financing, insurance and communication services.

By integrating previously separate fertility services under its online umbrella, the company disrupts the conventional clinic‐based delivery of fertility care and introduces new treatment rationales for reproductive decision‐making through its online and offline channels in line with its diverse portfolio. For example, the *Eeva* test had its own website, was the subject of expert advice on the *FertilityAuthority* platform and was introduced in moderator‐initiated discussions on the *FertileThoughts* forum. It was also included in Progyny's corporate fertility benefit package, which covered treatment plans for employees that ‘start with egg or embryo freezing, include testing of the embryo to reduce miscarriage, and include a single embryo transfer (SET) that when coupled with the healthiest embryo, result in the fastest track to success’ (McCarthy 2016). The *Eeva* test is thus embedded in a broader reframing of the reproductive process through Progyny's mission to ‘combine service, science and data to optimise the clinical outcomes of fertility treatments’ (Progyny 2017). Egg freezing is included as a means to avoid the risk of future involuntary childlessness and optimise a potential IVF procedure with higher quality eggs. The inclusion of data‐driven embryo selection approaches is rationalised as a condition for successful single embryo transfer to avoid multiple births.

Progyny's vision thus promotes a treatment rationale that expands the scope of IVF both by encouraging younger fertile women to pre‐emptively undergo infertility treatment and by increasing the number of treatment steps in each cycle to optimise clinical outcomes. This reframing of the reproductive process entails both the financialisation of reproductive risk and its proposed mitigation through a highly technologised and revenue‐generating set of treatments. Progyny's pre‐emptive treatment rationale of avoiding reproductive and financial risk thus normalises a high‐tech IVF treatment course for a larger group of potential candidates, which may be reached through both online platforms and their employers' HR departments, and unambiguously represents reproductive and data technologies as the best risk‐mitigating strategies to ‘ensure that anyone who wants to have a child, can have one’ (Progyny 2017).

### Consolidating the whole IVF journey

The increasing prevalence of time‐lapse embryo imaging also intersects with a consolidating trend of vertical integration of the fertility industry, as those companies producing reproductive data technologies expand their portfolios to cover the ‘entire IVF journey.’ All of the major companies producing time‐lapse imaging apparatuses – Genea, Progyny, Merck and Vitrolife – explicitly voice this ambition in their marketing and investment materials. Vitrolife's presents its various products – laboratory instruments, culture media, imaging technologies, etc – as an integrated portfolio to ‘maximise success every step of the way’ (Vitrolife 2018c). Likewise, upon introducing its Geri time‐lapse system, Merck announced that ‘With an Extended Fertility Technologies Portfolio Merck now Covers all IVF Steps’ (Merck 2016). After various acquisitions and alliances since 2013, Merck and Vitrolife, a pharmaceutical and biotechnological company, currently distribute all four major time‐lapse embryo imaging systems. In line with the ambition to cover every stage of the IVF process, the ‘add‐on’ technology of time‐lapse embryo imaging provides an opportunity to expand the treatment steps per cycle and popularise new forms of standardisation within assisted reproduction.

Vitrolife produces both the *Primovision* system and, after acquisition of its former producer Fertilitech in 2014, the *Embryoscope*. Vitrolife specialises in IVF culture media and disposables, such as pipettes and dishes. The inclusion of time‐lapse embryo imaging in their business model has proven to be highly successful and sales of these machines have increased each quarter since 2014 (Vitrolife 2018a). They estimate that 10% of IVF centres in the world and over half of UK clinics use their time‐lapse embryo imaging machines (Vitrolife 2017, p. 3). These high figures indicate that a growing number of patients and professionals will encounter the option to include these machines as part of their IVF cycles. As the company seeks to cover every step of the reproductive process, the IVF cycle overlaps with the ‘value chain’ of Vitrolife products (Axelsson 2016, p. 6). The added step of data‐driven embryo‐selection technology affirms the wider trend of the ‘value per cycle increasing through better technologies,’ an effect that is intensified by a related trend of more cycles following a single ‘oocyte pick‐up’ (Axelsson 2016, p. 16). Addressing investors, the company specifically makes the business case for time‐lapse embryo imaging as a high‐tech marketing tool and as a means to increase income per cycle, given that the cost (€50–€200) is significantly lower than the standard selling price (€400–€1000) per treatment (Ramsing 2016, p. 11). In considering digital reproductive health, it is important to highlight that the emergence of new digital subjectivities, knowledges and networks is situated in a rapidly growing global fertility sector; its rationality of expansion is a key driver of the increasing datafication of reproduction.

Another major player in the fertility sector is Merck, a multinational pharmaceutical company with approximately 50,000 employees in 70 countries, which is a leading distributor of the fertility drugs used in IVF cycles. Recently biosimilars to Merck's major fertility drugs for IVF ovarian stimulation have been introduced (Allahbadia and Allahbadia 2016, Winstel *et al*. 2017). At this time, the company is also expanding its portfolio to include time‐lapse embryo imaging by partnering with both Genea, which produces the *Geri* system, and abovementioned Progyny, which produces the *Eeva* test. Investments in these data‐driven systems are part of its broader strategy to ‘cover all IVF steps’ and develop ‘from a drug provider to an integrated fertility partner’ (Wenzel 2017). Alongside this ambition, a key goal of the Global Fertility Alliance, of which Merck is a founding member, is to promote ‘standardisation and automation in *in vitro* fertilisation (IVF) clinics’ (GFA 2018). Investments in automated and standardised embryo selection through data‐driven technologies that materialise these principles align with this wider goal.

The blurring of the lines between clinical and capitalist rationales in these global reproductive bioeconomies is foundational to the datafication of reproduction and raises concerns about the implications of the concomitant corporatisation of IVF. The valuation of time‐lapse embryo imaging follows not only from the commodification of add‐on treatments, algorithms and selection apparatuses, but it is also a materialisation of an expansive drive within global IVF enabled by standardisation, automation and data‐generativity. Alongside a critique that IVF becomes overly shaped by corporate interests is a concern that the specific corporatisation of data‐driven embryo selection may both enlist fertility clinics and patients in treatment rationales that require even more investment per cycle and create technological lock‐ins that make clinics beholden to particular platforms, thereby potentially intensifying data and financial asymmetries within assisted reproduction (Kitchin 2014, pp. 181–2).

## Conclusion

The data‐driven selection of embryos with time‐lapse embryo imaging has primarily been discussed in terms of its clinical efficacy, but its introduction reflects and reconfigures a range of practices within the contemporary fertility sector. As Sarah Franklin (2013) has argued for IVF, time‐lapse embryo imaging provides a lens onto the reconceptualisation and recommodification of prenatal life when data technologies and reproductive technologies meet.

The datafication of embryo selection shifts clinical practice by introducing a new treatment option that renders embryo viability visible and calculable by means of an algorithmically assisted way of seeing. With the introduction of this ‘*in silico* vision’ in the embryological workflow, IVF cycles do not only produce babies, but also sizable data sets on embryo development. As data flows of embryos are shared – or withheld – between embryologists, corporations and patients, embryo selection becomes a more networked and commercialised activity in which different actors have a stake.

The establishment of emergent reproductive data infrastructures through the introduction of growing numbers of time‐lapse embryo imaging systems raises questions about who may access and who can claim ownership of this embryo data. The patenting of this process highlights how the embryos’ data‐generativity may be repurposed as a method for selection, how observable characteristics of embryo development are transformed into private property, and how the development of bioinnovations is increasingly reliant on funding from for‐profit partners. The sizable data sets about embryogenesis collected through this system provide the basis for the creation of new algorithmic products for embryo selection by biotechnological and pharmaceutical companies. This process of turning biodata into biocapital relies on reproductive data infrastructures, through which new data and power asymmetries between different actors in the fertility sector are construed and consolidated.

What is remarkable about the commercialisation of time‐lapse technologies is the way in which strategies of patenting, direct‐to‐consumer branding, privately held data accumulation, its algorithmisation into selection tools and ownership of the whole IVF supply chain are combined into a total system for data‐driven embryo selection. This multipronged move towards datafication, and the concomitant promise of automation, standardisation and data/capital accumulation in a more networked mode of embryo selection, both reflects and reinforces a consolidating trend in the fertility sector – characterised by mergers resulting in larger fertility chains, online platforms adopting a key role in the organisation of fertility care and the portfolio expansion of pharmaceutical and biotechnological companies to cover each step of the IVF cycle.

In the context of this Special Issue I therefore want to emphasise that the emergence of new digital subjectivities, knowledges and networks in digital reproductive health are situated in a rapidly growing global fertility sector; its rationality of expansion is a key driver of the datafication of reproduction. What is at stake, then, in the enmeshed forms of biocapital and biodata that emerge with the datafication of (reproductive) health care is not only the increase or decrease of pregnancy rates, but numerous conceptual, epistemological and institutional shifts that lie at the foundation of both contemporary technologised reproduction and the future reconfigurations of the relation between biomedicine and society. It is, in other words, crucial to understand data‐driven IVF as not a peripheral phenomenon, but as a harbinger of how power relations between networks of social and corporate actors can be built‐in to the institutional infrastructures that deliver digital health.

## Notes

1 For a discussion of the medical gaze in relation to the prenatal imagery produced in time‐lapse embryo imaging, see Van de Wiel (2017, 2018).

2 Time‐lapse embryo imaging is widely used in scientific studies in developmental biology. The tripling of citations on time‐lapse embryo imaging from 447 to 1358 between 2013 and 2017 gives an indication of the impact of this technology on the field. Citation report generated at Thomson Reuter's Web of Science ‘([time‐lapse OR “time lapse”] AND [IVF OR ICSI OR “embryo selection”]).’

3 See Fox Keller (1996).

4 See Thompson (2013) for a detailed discussion of the Dickey–Wicker Amendment (pp. 79–84).

5 See Franklin (2013) for a discussion of the ‘retooling’ of reproductive substance in processes of ‘bioindustrialisation’ (p. 64).

6 With a network of over 455 clinics and a focus on servicing Fortune 500 companies, Progyny is currently the leading fertility benefits provider in the US.
